# A Case of Seronegative ANA Hydralazine-Induced Lupus Presenting With Pericardial Effusion and Pleural Effusion

**DOI:** 10.7759/cureus.9831

**Published:** 2020-08-18

**Authors:** Karim O Elkholy, Hamza Akhtar, Abishek Chakraborti

**Affiliations:** 1 Internal Medicine, Brookdale University Hospital Medical Center, New York, USA

**Keywords:** hydralazine, hydralazine induced lupus, ana, pericardial effusion, drug induced lupus, pleural effusion, negative ana, anti-histone, transthoracic echocardiography, pericardiocentesis

## Abstract

Hydralazine induced lupus syndrome (HILS), a form of Drug-Induced Lupus (DIL), was first reported in 1953. Since then, studies have shown an increasing incidence of HILS. It presents with lupus-like symptoms such as arthralgia, fever, chest pain, anorexia, fatigue, petechiae, and rash. Though rare, HILS may initially present with pericardial effusion. Lab findings of HILS usually show positive anti-nuclear antibody (ANA) in >95% of cases, antihistone abs in >95% of cases, rheumatoid factor ab in 20%, and anti-double-strand DNA in <5%. Herein we present a case of HILS which initially presented with a seronegative ANA and pericardial effusion. An 82-year-old woman who presented with shortness of breath was found to have bilateral pleural effusion and pericardial effusion. Common etiologies of pericardial effusion have been ruled out, after careful review of her home medications, hydralazine was suspected to be the culprit of her pericardial effusion. Initial ANA testing was negative, however given high clinical suspicion autoimmune disease screening was done revealing positive anti-histone antibodies. Hydralazine was deemed to be the etiology of her pericardial effusion which led to the discontinuation of the drug. Serial echocardiography revealed no recurrence of the effusion.

## Introduction

Hydralazine-induced lupus syndrome (HILS) is a form of drug-induced lupus (DIL). While the mainstay of diagnosing HILS is by positive screening serology of anti-nuclear antibody (ANA) and confirmatory antihistone antibodies in conjunction with a clinical picture consistent with the disease, it could be challenging to diagnose HILS in the settings of seronegative ANA. The association between ANA seronegativity and HILS could be associated with worse outcomes. Pericardial effusion is a serious condition and determining etiology could be challenging. DIL should remain a suspicion in patients with unclear etiology of pericardial effusion despite initial ANA seronegativity. We here present a challenging case of pericardial effusion related to HILS and initial ANA seronegativity.

## Case presentation

An 82-year-old woman presented to the Emergency Department (ED) with progressive shortness of breath that had started three days prior and was associated with bilateral swelling of the lower extremities. The patient also complained of orthopnea but did not notice any cough, fever, or chest pain. The patient denied any alcohol, tobacco, or illicit drug use but had a significant past medical history of type II diabetes mellitus, hypertension, hyperlipidemia, diastolic heart failure, peripheral arterial disease, hypothyroidism, and stage 3B chronic kidney disease (CKD). Home medications included amlodipine, benazepril, hydralazine, hydrochlorothiazide, aspirin, clopidogrel, cilostazol, atorvastatin, and basaglar insulin. At presentation, the patient had a blood pressure of 176/82 mmHg, a pulse of 78 bpm, a temperature of 98°F, a respiratory rate of 28 breaths/min, and an oxygen saturation in room air of 92%. Physical examination revealed reduced bilateral breathing sounds in the lung bases, distant heart sounds with a normal S1 and S2, no jugular venous distension (JVD), and bilateral pitting edema of the lower extremities up to the mid-calf.

**Figure 1:**
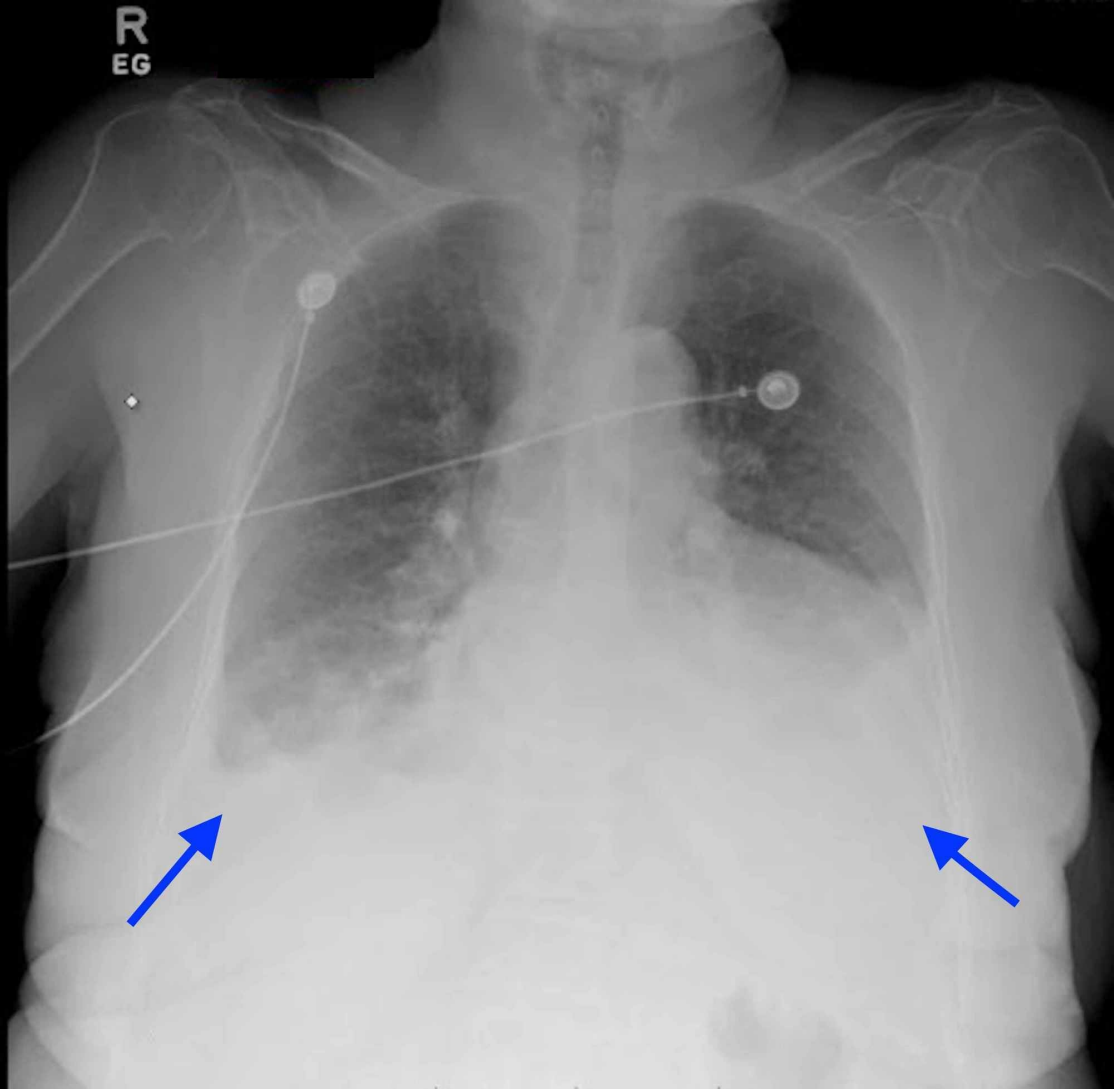
Chest X-ray shows bilateral pleural effusions (blue arrows) and cardiomegaly.

Initial testing showed: normocytic anemia with a hemoglobin (Hb) of 9 g/dL, CKD with a creatinine (Cr) of 1.86 mg/dL and a glomerular filtration rate (GFR) of 31, an elevated NT-pro B-type natriuretic peptide (PBNP) of 3760 pg/dL, an elevated D-dimer 3110 DDU, and negative cardiac enzymes. Chest X-ray showed cardiomegaly and bilateral pleural effusions [Figure 1]. 

**Figure 2:**
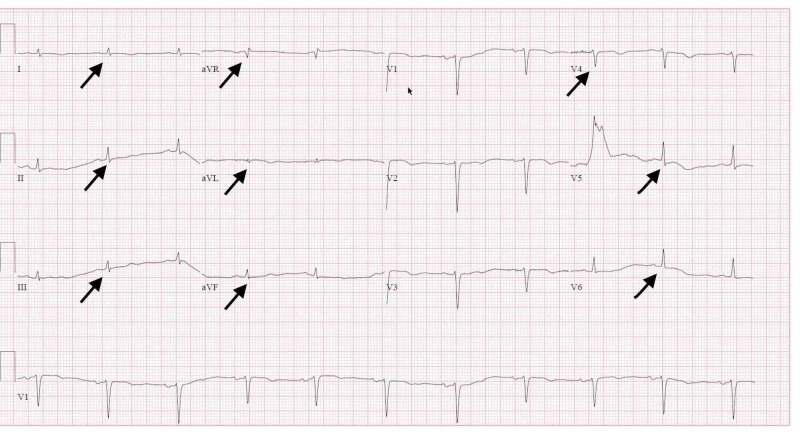
12 leads electrocardiogram (EKG) shows low voltage QRS (black arrows)

Electrocardiogram (EKG) at presentation showed low voltage QRS complexes in most leads, with nonspecific t-wave abnormalities, and the heart rate was 76 bpm in normal sinus rhythm [Figure 2].

**Figure 3:**
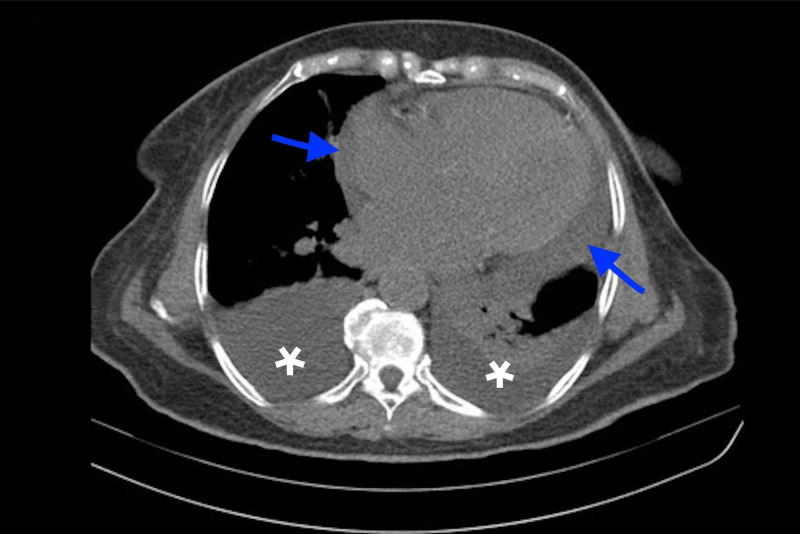
CT chest shows bilateral pleural effusions (white asterisks) and pericardial effusion (blue arrows).

Computed Tomography (CT) of the chest was done that showed cardiomegaly with pericardial effusion and bilateral pleural effusion [Figure 3]. 

The patient was started on furosemide for diuresis in the face of acute exacerbation of heart failure. She received one day of therapeutic anticoagulation in view of her high Wells score and D-dimer levels indicative of possible Venous thrombo-embolism. A pulmonary embolism was ruled out with a ventilation-perfusion scan. Lower extremity duplex showed no deep vein thrombosis.

**Figure 4:**
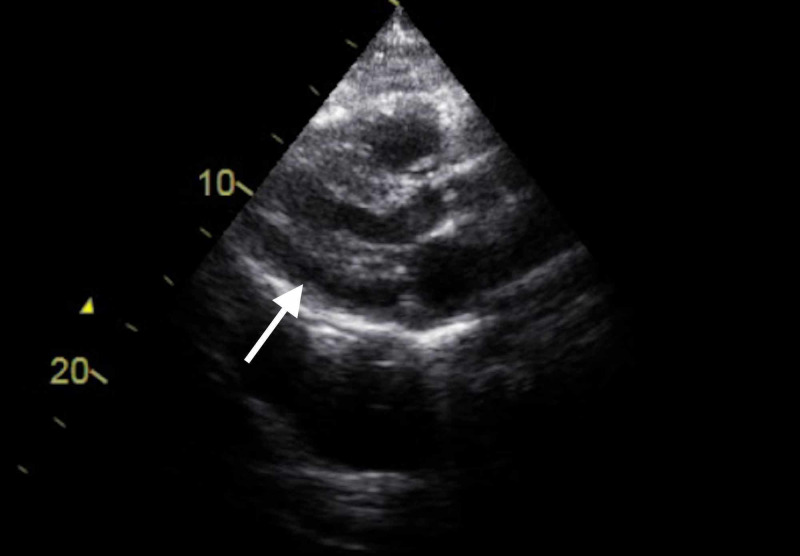
Initial echocardiography shows pericardial effusion (white arrow).

An official echocardiogram was performed and showed moderate pericardial effusion (13 mm) with EF 50-55% and diastolic dysfunction [Figure 4].

The previous echocardiogram from three months prior showed preserved left ventricular ejection fraction with grade 1 diastolic dysfunction and a trace pericardial effusion. It was decided to continue to monitor the effusion with serial echocardiograms as there was no clinical evidence of tamponade physiology. 

**Figure 5:**
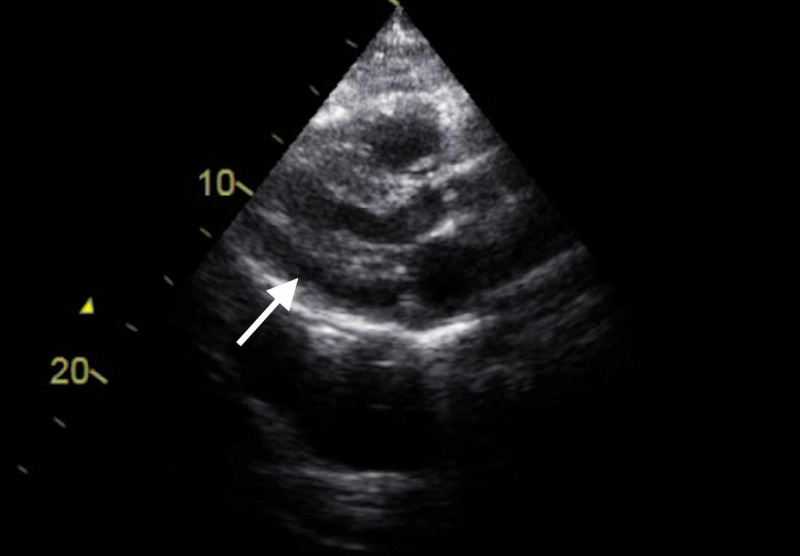
Echocardiography shows pericardial effusion (white arrow).

The patient's respiratory status mildly improved with continued diuresis. A repeat echocardiogram on the sixth day of hospitalization showed a persistent moderate pericardial effusion (measuring 13 mm) [Figure 5]. The patient remained hemodynamically stable with no evidence of tamponade physiology. 

After discussing options with the patient she agreed to a therapeutic as well as diagnostic pericardiocentesis. Straw-colored pericardial fluid (620 ml) was drained and a pericardial drainage catheter was left in-situ. Pericardial fluid analysis was suggestive of transudative fluid with albumin 2.7 g/dL pH 8, lactate dehydrogenase (LDH) 179 units, cytology was negative for malignant cells, and macrophages were present. Gram stains and cultures were unremarkable. 

The patient's thyroid stimulating hormone (TSH) and thyroxine (T3/T4) levels were within normal limits; her home dose of levothyroxine was continued and hypothyroidism seemed unlikely to be the precipitating factor for her pericardial effusion. 

Due to the high output from the pericardial catheter, further investigation was warranted to delineate the etiology of the effusion. Autoimmune diseases screening was done and results are shown in Table 1. 

**Table 1:** Laboratory tests for autoimmune diseases screening. ANA: anti-nuclear antibody; ANCA: anti-neutrophil cytoplasmic antibodies; DsDNA: double-stranded DNA; CCP: cyclic citrullinated peptide; SSA/SSB: Sjogren Syndrome A, Sjogren Syndrome B; Scl-70: Scleroderma-70; Ab: antibodies

Laboratory tests	Initial	Repeat
ANA	Negative	Negative
ANCA	Negative	-
DsDNA Ab.	Negative	-
CCP Ab.	Negative	-
Anti-smith Ab.	Negative	-
SSA/SSB Ab.	Negative	-
Scl-70 Ab.	Negative	-
Anti-histone Ab.	1.5 (positive)	1.9 (positive)

A diagnosis of pericardial effusion secondary to HILS was made and hydralazine was stopped indefinitely. A repeat echocardiogram a week after stopping the medication showed no pericardial effusion and normal left ventricular ejection fraction. Over the course of her hospital stay the patient developed a left middle cerebral artery ischemic infarct and was discharged to a rehabilitation facility.

## Discussion

DIL has been associated with more than 70 medications, with hydralazine being the most commonly reported [1]. Hydralazine use is fairly prevalent following the African American Heart Failure Trial that demonstrated mortality benefit with the use of BiDiL (hydralazine and isosorbide dinitrate) [2]. The use of hydralazine has been growing steadily since then [3]. HILS was first reported in 1953 which described a patient with rheumatoid arthritis-like clinical picture associated with the use of hydralazine [4]. Since then, studies have shown an increasing incidence of HILS in patients taking hydralazine; one study reported the incidence of HILS to be around 6.7% of patients taking hydralazine [5]. The incidence of HILS was dose-dependent; 5.4% at a dose of 100 mg daily and 10.4% at a dose of 200 mg daily [5]. However slow acetylators can develop toxicities at lower doses [6]. The pathophysiology of HILS can be explained by the fact that hydralazine decreases T cell DNA methylation by inhibiting the extracellular signal-regulated kinase (ERK) pathway which mimics the defects found in lupus T cells [7]. Manifestations of HILS includes arthralgia/myalgia (80%), fever/weight loss/fatigue (40-50%), hepatosplenomegaly (15%), pericarditis (<5%), rash (25%), glomerulonephritis (5-10%), pleuritis/pleural effusion (<5%) and neuropsychiatric symptoms (<5%) [8]. Lab findings of HILS include presence of ANA in more than 95% of cases , antihistone antibodies in more than 95% of cases, rheumatoid factor Ab in 20% of cases, and anti-double-strand DNA in less than 5% of cases [9].

The association between ANA-negative HILS and pericardial effusion has been described in the literature. We provided, to the best of our knowledge, cases that have been reported with similar presentations [Table 2].

**Table 2:** Case reports describing DIL with pericardial effusion and/or ANA seronegativity. ANA: anti-nuclear antibody; DIL: drug-induced lupus

Authors	ANA	Dug used	Pericardial effusion
Iyer P., et. al. [10]	Negative	Hydralazine	Present
Vicken Zeitjian et. al. [11]	Negative	Hydralazine	Present
Tahir, M et. al. [12]	Negative	Hydralazine	Present
Chamsi Pasha, et. al. [13]	Positive	Hydralazine	Present
Ajakumar Menon et. al. [14]	Positive	Hydralazine	Present*
Carter, et. al. [15]	Negative	lisinopril	Not present
Solomon-Tsegaye et. al. [16]	Negative	Hydralazine	Not present

The mainstay treatment of HILS includes the identification of the drug as the cause of the pathology and stopping it, usually symptoms resolve within weeks of discontinuing the drug [17]. Non-Steroidal Anti-inflammatory Drugs (NSAIDs) or low dose corticosteroids may be considered for mild manifestations, with high dose corticosteroids reserved for more serious manifestations [17].

## Conclusions

Our case describes a patient with uncommon manifestations of HILS including pleural effusion and pericardial effusion with negative ANA serology. The association between ANA-negative HILS and pericardial effusion deserves further research because it could be associated with worse outcomes. While initial ANA seronegativity can mislead clinicians in diagnosing HILS, pericardial effusion secondary to HILS should remain a suspicion in any patient taking hydralazine. Treatment usually involves stopping the drug and following up the effusion with echocardiography to ensure there is no recurrence.
